# Special Issue: Integrated Pest Management in Arable and Open Field Horticultural Crops

**DOI:** 10.3390/insects11020082

**Published:** 2020-01-23

**Authors:** Andrew G. S. Cuthbertson

**Affiliations:** Independent Science Advisor, York YO10 5AQ, UK; andrew_cuthbertson@live.co.uk

**Keywords:** arable crops, horticulture, integrated pest management, pesticides

## Abstract

Invertebrate pest control within both agricultural and horticultural production systems continues to present many challenges. Over the past decades the commonly used method for pest control has been the direct application of chemical products. However, in response to environmental, economic, and other problems associated with the over-reliance on chemical insecticides there has been an increasing drive towards the development of Integrated Pest Management (IPM) approaches. Many IPM strategies are now well developed under protected environments. However, within the open field in many situations targeted success is yet to be achieved. This special issue will seek to showcase original articles and reviews by leading research entomologists and associated experts. Articles presented will focus on the development and implementation of IPM strategies against various major arable and horticultural invertebrate pests (both indigenous and invasive species).

For the past few decades the control of injurious invertebrate pests within cropping systems has been largely undertaken by the direct use of broad-spectrum chemical products. These have no doubt over the years provided excellent control of pest species and have contributed greatly to the increased crop yields and productivity that has been experienced globally. However, the impact of the prophylactic use of such products on biodiversity, ecosystems and their wider services worldwide went largely unnoticed. Now with an increasing awareness of environmental, economic, and other problems associated with the over-reliance on chemical pesticides there has been an increasing drive towards the development and implementation of Integrated Pest Management (IPM) approaches. Indeed, the prophylactic use of broad-spectrum insecticides has been attributed to the ‘formation’ in certain cases of what are now classed as major horticultural pests. The concept of IPM simply means giving careful consideration to all available plant protection products and seeking their integration in order to control a given pest species. Chemical products are not ‘banned’ from IPM strategies, but their application timing is carefully considered in order to have most impact on the pest and least impact on non-target organisms and the wider environment.

IPM is therefore not a single pest control strategy. It is a combination of pest management evaluation, risk decision making and various control options. Generally, farmers/growers who aim to practice IPM would undertake the following procedures:

**Identify and Monitor Pests:** Not all insects that occur on a given cereal crop or horticultural fruit tree are a pest and require control. Correct identification of what is a pest and what is beneficial is extremely important to avoid unnecessary chemical applications. Many insects occurring on a crop are innocuous, and indeed may even provide a beneficial role. IPM therefore requires effective identification and monitoring of pest issues. A thorough understanding of a species biology, population development, most damaging life stage, and the most susceptible life stage to given control options is critical for determining the most efficient control strategy to employ. Accurate monitoring minimises the potential for interventions to be applied when they are not needed, and alternatively is used to ensure the correct timing of the control method (chemical or non-chemical) in order to optimise effectiveness.

**Setting of Action Thresholds:** The first step before any form of pest control is applied is the determination of an ‘action threshold’. This is the point that when pest population numbers are reached, or the prevailing environmental conditions dictate control action must be applied in order to maintain crop economic productivity. The occurrence of pest individuals on a crop does not necessarily mean that action needs to be taken. The emphasis is on control rather than complete eradication of a pest species. The concept behind IPM is that a low surviving pest population is important to provide a food source for natural predators, which can then continue to prevent significant population development during the cropping season.

**Prevention:** The first line of crop defence against a pest is prevention where possible. IPM strategies are designed to manage a given crop to aid in preventing a given pest species from becoming a problem. Methods employed to aid pest prevention include rotating between different crops, selecting pest-resistant varieties, planting pest-free rootstock and the maintenance of good crop sanitation.

**Control:** Only when correct identification, monitoring, and the action thresholds indicate that pest control is required, and the preventive methods are no longer effective or available, do other control methods need to be applied. In this process IPM strategies will evaluate a potential control option for both its effectiveness and wider environmental risk. Ideally, low risk control options are applied first, such as mechanical control, for example trapping, physical weeding, or the release of biological control agents. Targeted chemical control using pheromones to disrupt insect mating or within ‘attract and kill’ traps can also prove effective. Should such methods of mild control not provide sufficient efficacy then additional options need to be employed and this may involve targeted spraying of chemical insecticides. However, broad spectrum spraying of non-specific insecticides is not encouraged.

The increasing concern over the continued use of chemical pesticides on the environment is pressurising arable and horticultural producers to look for alternatives to chemical pest control. Increasing consumer awareness in relation to chemical residues on food produce and harmful effects on non-target species as well as a continual loss of active ingredients and increasing reports of target pest resistance is also driving the need for alternative means of invertebrate pest control to be devised. The concept of pest control has now changed to pest management as a balanced approach to managing pest populations to levels that do not cause economic damage. This is favoured over total eradication (except in the case of an introduced invasive species) for both financial and environmental reasons. The continued development and implementation of IPM strategies is therefore essential in order to manage invertebrate pests without total dependence on chemical pesticides and therefore contribute to the maintenance of sustainable cropping ecosystems.

